# Benign intestinal glandular lesions in the vagina: a possible correlation with implantation

**DOI:** 10.1186/s13000-016-0503-5

**Published:** 2016-06-17

**Authors:** Weiwei Lu, Xiaofei Zhang, Bingjian Lu

**Affiliations:** Department of Surgical Pathology, Department of Gynecologic Oncology, Women’s Hospital, School of Medicine, Zhejiang University, 1 Xueshi Road, Hangzhou, 310006 Zhejiang Province People’s Republic of China; Department of Surgical Pathology, the Affiliated Women’s Hospital, School of Medicine, Zhejiang University, 1 Xueshi Road, Hangzhou, 310006 Zhejiang Province People’s Republic of China

**Keywords:** Vagina, Polyp, Adenosis, Intestine, Cloacogenic

## Abstract

**Background:**

Enteric-type glandular lesions are extremely rare in the vagina. Their histological origin remains a matter of speculation at present.

**Method:**

We review two rectal mucosal prolapse-like polyps and one intestinal-type adenosis in the vagina.

**Results:**

Case 1, a 64-year-old woman, presented with a vaginal polypoid lesion with a size of 4 × 3 × 3 cm. Case 2, an 8-year-old girl, had a 1.5 × 1.5 × 0.8-cm pedunculated polyp in the vaginal navicular fossa and a clinically suspected rectovaginal fistula. Case 1 and 3 had an obsolete severe perineal laceration. On histopathological examination, cases 1 and 2 resembled rectal mucosal prolapse or inflammatory cloacogenic polyp (rectal mucosal prolapse-like polyp). Case 3 had an incidental intestinal-type adenosis in the removed vaginal wall. Immunohistochemistry confirmed the intestinal differentiation in all 3 lesions by showing diffuse CDX2-positive, CK20-positive, and scattered chromogranin A-positive neuroendocrinal cells in the lower compartment of the crypt.

**Conclusions:**

In summary, we report herein three unusual cases of benign intestinal-type glandular lesions in the vagina including two rectal mucosal prolapse-like polyps and one case of intestinal-type adenosis, and discuss possibilities for their histogenetic basis.

## Background

Adenomas and adenocarcinomas with intestinal differentiation have rarely been reported in the vagina [1–4]. The origin of these enteric-type glandular tumors has remained the subject of speculation because non-neoplastic intestinal epithelium is exceptionally rare either in pure forms or the vicinity to the tumors [4, 5]. In this report, we describe 3 unusual cases with non-neoplastic intestinal glands: 2 vaginal polyps resembling rectal mucosal prolapse and 1 intestinal-type vaginal adenosis.

## Methods

Three cases of benign non-neoplastic lesion with intestinal glands in the vagina were retrieved from the electronic archives of the Department of Surgical Pathology, the Affiliated Women’s Hospital School of Medicine Zhejiang University, China, over a period between 2007 and 2014. This study was approved by the Institutional Research Ethics Committee of the Affiliated Women’s Hospital School of Medicine Zhejiang University, China. The patients or her parents (case 2) were well informed and consent with this study. We obtained clinical details and follow-up data from the hospital medical records. Archival routine H&E slides were assessed by the authors.

Immunohistochemistry was carried out using the properly diluted antibodies against CK7 (OV-TL, 1:100), CK20 (KS20.8. 1:100), CDX2 (DAK-CDX2, 1:100), PAX8 (ZR1, 1:50), GATA3 (1:100) and chromogranin A (IR502, 1200). A two-step En Vision immunostaining procedure (DAKO, Carpentaria, CA, USA) was performed according to the manufacturer’s protocols.

## Results

The clinicopathological features of these cases are summarized in Table 1.

**Table 1:** Clinicopathological features of benign glandular lesions in the vagina

	Case 1	Case 2	Case 3
Age (yr)	64	8	52
Gestation & parity	G2P2	G0P0	G4P3
History of disease	Severe laceration	No	Severe laceration
Clinical presentation	An incomplete incontinence of feces	Vaginal bleeding	An incomplete incontinence of feces
Vaginal Location	Lower posterior wall	Posterior wall	Lower posterior wall
Surgery	Vaginal polypectomy; LEEP	Vaginal polypectomy	Removal of the endometrial polyp and leiomyoma; repair of the perineal laceration and the posterior vaginal wall
Gross findings (size)	Polypoid mass (3.5*2.5*1.0 cm)	Polypoid mass (1.5*1.2*0.5 cm)	Unremarkable vaginal wall (3*2*0.4 cm)
Histopathological findings	Rectal mucosal prolapse-like polyp	Rectal mucosal prolapse-like polyp	Intestinal-type adenosis
Other findings	CINII	Rectovaginal fistula	Endometrial polyp; submucosa leiomyoma

**Fig. 1:**
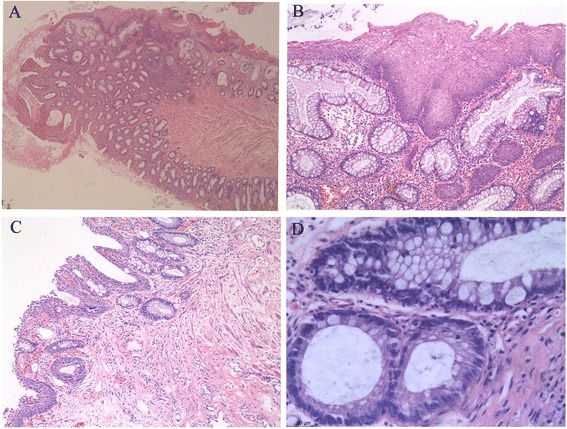
Intestinal glands in the benign vaginal lesions. The polypoid lesions in case 1 and 2 are histologically identical to that of colorectal mucosal prolapse **a. b** depicts the presence of squamous epithelium in the polyps. The transitional epithelium mimicking “anal transformational zone” in the rectal-anal canal junction is shown in **c**. Intestinal glands from case 3 is given in **d**. (H&E staining, original magnifications: *A* 2.5*10; *B,C,D* 10*10)
    - The “muscularis mucosa” in the polyps was thickened and somewhat different from that in the rectum by showing a disordered arrangement of smooth muscles. Some smooth muscle fibers may protrude into the mucosa proper and separate the intestinal glands. The “submucosa layer” contained loose fibrous tissue, fibro-adipose tissue and focal clusters of dilated lymphatic vessels.
    - The LEEP specimen in case 1 had focal CIN II with glandular involvement and clear margins.
  - Case 3
    - The removed lower posterior wall of the vagina measured 3.0 × 2.0 × 0.4 cm. It was grossly unremarkable. A small cluster of “intestinal-type” glands in the lamina proper were incidentally found in routine slides. They were composed of predominant columnar cells with brush borders and scattered goblet cells with a single large mucin-containing vacuole [Fig. 1d]. The glands showed no evidence of dysplasia. The surface squamous epithelium showed a transition into the clonic type glands. Paneth cells, squamous metaplasia, and endocervical glands of common adenosis were not identified on routine stained slides. Mild inflammatory cell infiltration was found in the lamina proper. The endometrial polyp and leiomyoma were also histologically confirmed.
- Immunohistochemical findings
  - Immunohistochemical staining showed that the intestinal glands in all cases were positive for CK20 and CDX2 [Fig. 2a, c], and negative for CK7, GATA3 and PAX8. Neuroendocrinal cells in the intestinal glands were demonstrated by positive chromogranin A staining [Fig. 2b, d]. They were predominantly distributed in the lower compartment of the crypt. The squamous epithelium was negative for all these markers.

- Clinical Findings
  - Case 1
    - A 64-year-old Chinese woman, gravida 2 para 2, transferred to our hospital in February 2012 because she was diagnosed as cervical intraepithelial neoplasia grade II (CINII) by biopsy recently. During her admission, a reddish, soft, sessile polypoid mass was found in the lower posterior wall of the vagina (near the vaginal orifice). The polyp dropped from the vagina to the anus along the perineal skin. It was completely free from the vulvar skin and the anus. The smooth surface of the mass looked like the colorectal mucosa grossly. It measured 4.0 × 3.0 × 3.0 cm. The vulvar skin had an obsolete severe (Grade III) perineal laceration. The uterus and the uterine cervix looked unremarkable. Anal examination showed the decreased contraction capacity. The patient had undergone severe perineal laceration 45 years ago in the vaginal delivery of her first child, which resulted in an incomplete incontinence of feces. The patient was treated with a loop electrosurgical excision procedure (LEEP) of the uterine cervix and a vaginal polypectomy. She remained uneventful after her surgery.
  - Case 2
    - An 8-year Chinese girl presented with vaginal bleeding for 2 days. Her mother denied the use of diethylstilbestrol (DES) during pregnancy. Clinical examination found a pedunculated polyp in the posterior vaginal wall of the navicular fossa beneath the hymen. It measured 1.5 × 1.5 × 0.8 cm. Anal examination showed that the polyp was close to the right-anterior side of the rectum. The rectal mucosa was felt to be rough and cicatrical at the site of 1 cm from the anus. A rectal fistula to navicular fossa (rectovaginal fistula) was clinically suspected, but colonoscopy and colposcopy were not performed. The pelvic sonography was unremarkable. She underwent a polypectomy of the vagina. The suspicious rectovaginal fistula remained untreated because of her young age and potential diagnostic pitfalls by physical examination alone. She recovered well from the surger. She has been free of symptoms for 2 years at present.
  - Case 3
    - A 52-year Chinese woman, gravida 4 para 3, complained of an incomplete incontinence of feces after the severe perineal laceration more than 30 years ago in the vaginal delivery of her first child. She had no history of topical 5-fluorouracil use in the vagina. Gynecological examination showed an old severe (Grade III) perineal laceration at 12 o’clock which was involved the rectum. The sonography indicated the presence of a uterine endometrial polyp and a submucosa leiomyoma. She underwent hysteroscopic surgery to remove the endometrial polyp and leiomyoma, and repair of the perineal laceration and the posterior vaginal wall.
- Pathological Findings
  - Case 1 & 2
    - The vaginal polyp measured 3.5 × 2.5 × 1.0 cm and 1.5 × 1.2 × 0.5 cm in case 1 and case 2, respectively. Both polyps had a smooth surface. The cut surface was red, soft and edematous. The LEEP specimen in case 1 measured 1.0 × 3.0 × 1.0 cm and looked unremarkable grossly.
    - Both case 1 & 2 showed consistent histopathological features. Their histology resembled to that of a colorectal mucosal prolapse characterized by the surface “colonic-like mucosa” and the underlying “mucularis” [Fig. 1a]. The “colonic mucosa” manifested as elongated, distorted intestinal-type crypts and glands. Superficial erosions and inflammatory exudative were also focally present. A prominent lymphoplasmacytic infiltration with occasional lymphoid nodules was present in the mucosa. In addition to the glandular component, squamous epithelium was also seen [Fig. 1b]. The transitional pattern between glandular and squamous epithelium was morphologically identical to that of so called “anal transformational zone” in the rectal-anal canal junction [Fig. 1c]. There was no evidence of dysplasia in the polyps.

**Fig. 2:**
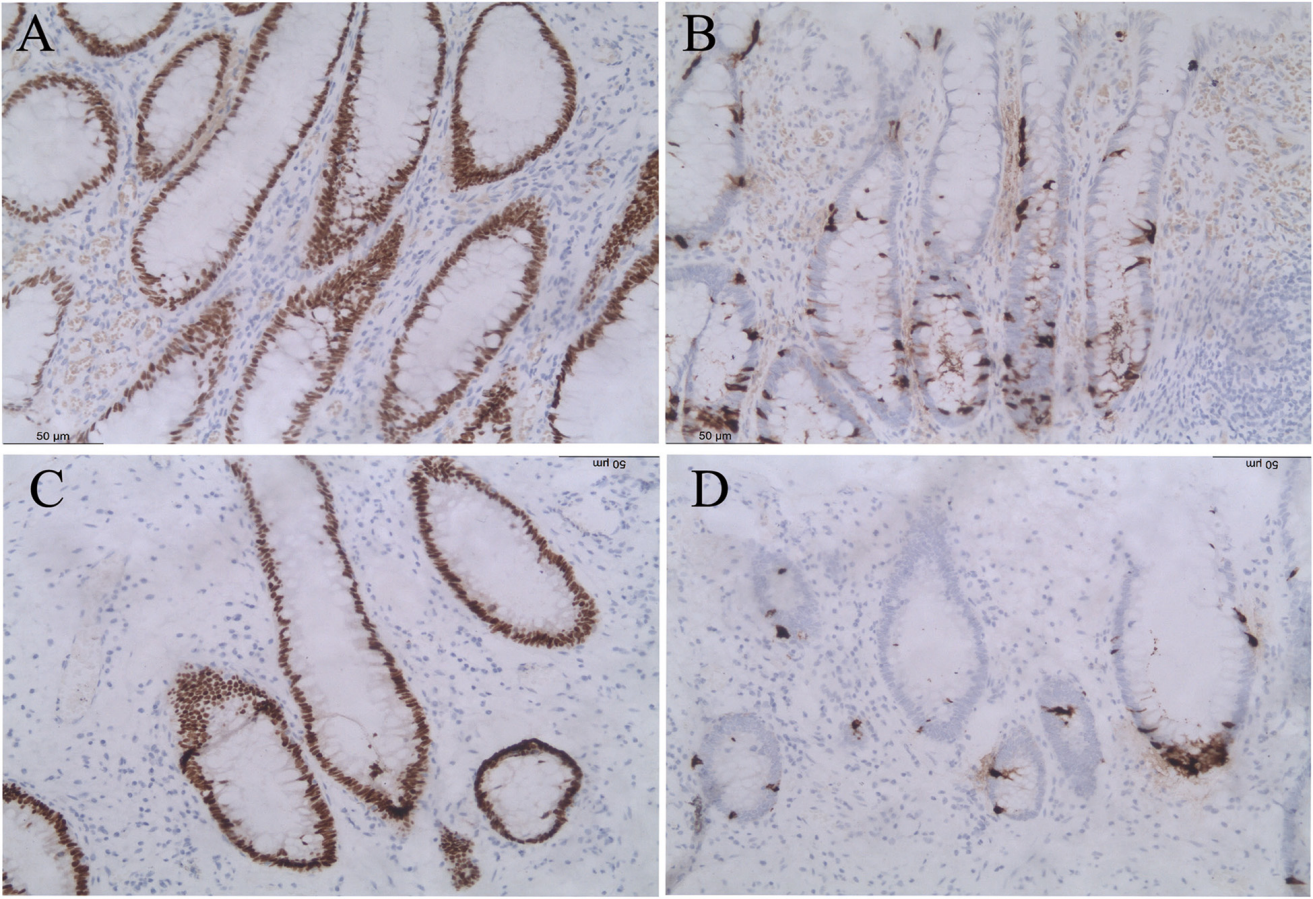
Immunohistochemical results of the intestinal glands in the benign vaginal lesions. Depicted is strong CDX2 positivity (**a**, **c**) and chromogranin A + ve neuroendocrine cells (**b**, **d**) in the intestinal glands. (*A*, *B*: case 1; *C*, *D*: case 3; Original magnifications: 20*10)

## Discussion

Intestine-type lesions are very uncommon in the vagina. They have a spectrum from benign non-neoplastic lesions to adenomas and adenocarcinomas. Only two non-neoplastic intestinal lesions, one polyp and another adenosis, have been documented in the vagina by now [4, 5]. In this report, we described two unusual polyps (case 1 and 2) and one adenosis (case 3) with intestinal-differentiation. All cases occurred in the lower posterior wall of the vagina, the most common region for intestinal-type adenocarcinomas [4]. The intestinal differentiation in our cases was characterized by goblet cells and a specific intestinal immunoprofile (strong CDX2-positive and CK20-positive), and absence of Müllerian (CK7-negative and PAX8-negative) or Wolffian (GATA3-negative) immunophenotype. Chromogranin A-positive neuroendocrinal cells were identified in the lower part of the crypts as in the colorectal mucosa.

Our polyps (case 1 & 2) were histologically identical to those of rectal mucosal prolapse, inflammatory cloacogenic polyp or solitary rectal ulcer syndrome. They had prominent smooth muscles, a feature distinct from the only intestinal polyp reported previously [4]. Therefore, they may be best characterized by the term “rectal mucosal prolapse-like polyp”. They should be differentiated from other benign glandular lesions. Rectal duplication, a rare developmental anomaly, usually presents as a cystic or tubular lesion in the rectum. Rarely, the extrophic subtype can be suspected as a “vaginal growth” [6]. However, rectal duplication does not communicate with vagina and rectum. It is consistent with the rectal histology, but the muscular layer is usually not well formed. Moreover, rectal duplication is commonly associated with other urinary and vertebrate malformations. Intestinal adenomas and juvenile polyps have been found in the vulvar and vaginal region [1, 3, 4, 7–10]. Both lesions do not show the structure of the rectal mucosa and muscular layer. Adenomas typically harbor varying degrees of dysplasia. Juvenile polyps are characterized by dilated cystic glands containing mucin in the background of inflamed, edematous granulation tissue. Adenosis manifests as the red granular spots or patches in the vaginal mucosa. The glandular or metaplastic epithelium replaces the original vaginal squamous epithelium. They look like the normal endocervical mucosa, with extremely rare intestinal differentiation [5]. Both tubulo-squamous polyp and fibroepithelial polyp lack intestinal glands and prominent smooth muscles. Limited goblet cells can occasionally be present in tubulo-squamous polyp [11].

Vaginal adenosis, a lesion associated with diethylstilbestrol (DES) exposure in utero, has been described for more than 100 years. The glands of vaginal adenosis are of the Müllerian-type (endocervical and/or tubuloendometrial glands). Our case 3 had distinct intestinal differentiation. To the best of our knowledge, only one case of vaginal adenosis with intestinal metaplasia has been reported in the English literatures by now [5]. Unlike that case, our case did not have common endocervical-type glands and denied DES exposure in utero. The term “intestinal-type adenosis”, rather than “adenosis with intestinal differentiation”, is more appropriate for this case.

Primary vaginal intestinal-type mucinous adenocarcinoma is extremely rare [2, 4, 8–10, 12–14]. Their histologic origin remains largely elusive; possibilities include: 1) the cloacal remnants or heterotopic intestinal epithelium during the embryogenesis; 2) intestinal metaplasia from glandular epithelium in vaginal adenosis, endometriosis and Skene duct, etc.; 3) derivation from the colon or rectum secondary to vaginorectal fistula or other processes [4]. Our cases may shed some new lights on these interpretations.

The intestinal polyp-like lesions in case 1 & 3 are more likely to be associated with perineal laceration, implicating an “implantation” of rectal mucosa during the repair of laceration. A recent report showed that a vaginal intestinal type adenocarcinoma with a history of severe laceration harbored an area similar to the anorectal junction [14]. Another possibility of intestinal metaplasia in case 3 raised due to its close relationship with the surface mature squamous epithelium. In fact, intestinal metaplasia can occur in vaginal adenosis [5] as in the Müllerian glands of the ovary, uterus and uterine cervix [15–17]. Moreover, the presence of lymphocytic aggregate in this case also supported the postulation that inflammation may serve as a possible contributing factor in this process [5].

The girl in case 2 had a suspected rectovaginal fistula. Her polyp likely represented for another “implantation” of intestinal mucosa via the potential fistula. However, due to the unavailability of colonoscopy and colposcopy, and absence of related symptoms in the following 2 years, the rectovaginal fistula can not be fully validated. The possibility of other anorectal malformations should also not be ruled out accordingly. A retrospective study demonstrated that the majority of rectovaginal fistula may actually be other anorectal malformations, particularly cloacal remnants [18]. Cloacal remnants are the regions incorporating anorectal tissue into the posterior vaginal wall during the division process of cloaca [19]. Given the site and morphological features, the cloacal remnants may be another possible, but less likely origin of this intestinal polyp.

## Conclusion

In summary, we reported three unusual cases of benign intestinal-type glandular lesions in the vagina including two rectal mucosal prolapse-like polyps and one case of intestinal-type adenosis. Histogenetic possibilities were discussed. Understanding the latter may ultimately help to illuminate the histogenesis of vaginal adenocarcinomas.

## Abbreviations

DES, diethylstilbestrol; LEEP, loop electrosurgical excision procedure.
